# No time to rest: How the effects of climate change on nest decay threaten the conservation of apes in the wild

**DOI:** 10.1371/journal.pone.0252527

**Published:** 2021-06-30

**Authors:** Mattia Bessone, Lambert Booto, Antonio R. Santos, Hjalmar S. Kühl, Barbara Fruth

**Affiliations:** 1 School of Biological and Environmental Sciences, Liverpool John Moores University, Liverpool, United Kingdom; 2 LuiKotale Bonobo Project, Centre for Research and Conservation, Royal Zoological Society of Antwerp, Antwerp, Belgium; 3 German Centre for Integrative Biodiversity Research (iDiv) Halle-Jena-Leipzig, Leipzig, Germany; 4 Faculty of Biology/Department of Neurobiology, Ludwig Maximilians University of Munich, Planegg-Martinsried, Germany; 5 Department for the Ecology of Animal Societies, Max Planck Institute of Animal Behavior, Konstanz, Germany; USDA Forest Service, UNITED STATES

## Abstract

Since 1994, IUCN Red List assessments apply globally acknowledged standards to assess species distribution, abundance and trends. The extinction risk of a species has a major impact on conservation science and international funding mechanisms. Great ape species are listed as Endangered or Critically Endangered. Their populations are often assessed using their unique habit of constructing sleeping platforms, called nests. As nests rather than apes are counted, it is necessary to know the time it takes for nests to disappear to convert nest counts into ape numbers. However, nest decomposition is highly variable across sites and time and the factors involved are poorly understood. Here, we used 1,511 bonobo (*Pan paniscus*) nests and 15 years of climatic data (2003–2018) from the research site LuiKotale, Democratic Republic of the Congo, to investigate the effects of climate change and behavioural factors on nest decay time, using a Bayesian gamma survival model. We also tested the logistic regression method, a recommended time-efficient option for estimating nest decay time. Our climatic data showed a decreasing trend in precipitation across the 15 years of study. We found bonobo nests to have longer decay times in recent years. While the number of storms was the main factor driving nest decay time, nest construction type and tree species used were also important. We also found evidence for bonobo nesting behaviour being adapted to climatic conditions, namely strengthening the nest structure in response to unpredictable, harsh precipitation. By highlighting methodological caveats, we show that logistic regression is effective in estimating nest decay time under certain conditions. Our study reveals the impact of climate change on nest decay time in a tropical remote area. Failure to account for these changes would invalidate biomonitoring estimates of global significance, and subsequently jeopardize the conservation of great apes in the wild.

## Introduction

In the past 50 years, a marked increase in global mean temperature due to anthropogenic-induced climate change has affected tropical rainforests inhabited by the great apes, orangutans (*Pongo spp*.*)*, gorillas (*Gorilla spp*.*)*, chimpanzees (*Pan troglodytes subspp*.*)* and bonobos (*Pan paniscus*) [1–3]. The rise in mean temperatures has induced a reduction in average precipitation in many areas of the tropics [3–5], increased the length of the dry season [6–8] and disrupted the very functionality of rainforests worldwide [9, 10]. As such, climate change poses a threat to the conservation of great apes, animals that are highly endangered, with all species and subspecies currently classified as Endangered or Critically Endangered in the Red List of Threatened Species [11].

For effective monitoring and conservation of remaining populations, conservationists must have accurate knowledge of the size of these populations [12]. Great apes live at low densities and are difficult to observe directly in their habitat. However, they are unique among the non-human primates in that all weaned individuals, independent of sex and age [13], build structures called “sleeping platforms,” “beds” or “nests”. The capability of building nests is learned rather than innate [14], with infants and occasionally juveniles sharing night nests with their mothers. Usually, the nest foundation is composed of a strong side branch, with smaller branches and twigs shaped over it, forming an oval, nest-like structure to accommodate a sleeping ape at night [15], and sometimes for resting during day (i.e. day nests) [16]. New nests are built every night; reuse of previously constructed nests occurs where construction material is limited (e.g. [17]) but is relatively rare [16]. Although sometimes built on the ground [18, 19], particularly in gorillas [20], nests are commonly constructed in trees. Several hypotheses for nest building in great apes have been proposed [16], including increased comfort and sleep quality [21–24], enhanced thermoregulation [25, 26], reduced predation risk [27, 28] and insect avoidance [29, 30]. Nest building in great apes is thought to have been a crucial component in hominid evolution [16]. Today, nest counts are used as proxies for assessing great ape population density and abundance in the wild, and thus are considered as an important conservation tool. As nests persist for long in the forest and are easily observed by the human eye [31], nest counts, rather than ape counts, have become the gold standard for monitoring presence/absence, abundance and density of apes in the past 40 years [32–35]. Methods such as Standing Crop Nest Counts (SCNC) [32], in combination with Distance Sampling [36], have provided accurate density estimates requiring only a single visit to the field. However, there are some disadvantages. Nest production rate [37–39] and nest decomposition or nest decay time [40, 41] (i.e. conversion factors) are highly variable across species, space and time, but are required to scale down the number of counted nests to the number of apes [35], in order to permit estimations of great apes [42]. Nest survey methods not requiring the use of conversion factors have been proposed [43], but they demand multiple visits to the survey site, which is often hard to implement. More recently, the advent of camera traps [44] has allowed an estimation of great ape abundance via the remote observation of individuals [45, 46]. However, camera-trap methods have not yet been fully validated and they require costlier equipment than nest count methods. Thus, nest count surveys remain highly relevant, with SCNC being the most commonly used method for estimating ape density in the wild, both via traditional ground [47–50] or aerial [51, 52] line transects. Finally, to investigate population trends, a crucial body of information in conservation science, it is necessary to compare estimates of ape population density obtained at different points in time [53–56]. It is generally recommended to apply site- and time-specific conversion factors to data generated from a particular survey [35], ideally obtained by monitoring large samples of nests (representative of the survey period) until full decomposition [35]. However, as such studies could last several months, more time-efficient methods have been developed, such as the retrospective estimation of nest decay with a single revisit of a marked nest, using logistic regression [57], or hidden Markov chain [58] analysis. Nevertheless, with the exception of some authors who have modelled decomposition time across sites [53, 54], published decay times have been applied to different sites [34, 56, 59–61] and/or time periods [51, 62]. This is problematic, as nest decomposition may differ with differing climate conditions, leading to inaccurate population density estimates [56, 63]. Nest-specific factors are known to drive nest decay; for example, nests built at the same time by members of the same group of apes exhibit different decomposition times [40]. However, rainfall is often reported as the most important variable affecting nest decomposition time, with lower rainfall resulting in longer decay times [38, 40, 41, 64]. Therefore, a drier climate would be expected to increase the time for which great ape nests would remain visible in the forest [40]. This knowledge has serious implications for great ape conservation. When using SCNC, applying an inappropriately short nest decay time would produce a falsely high population density result [42]. Therefore, if we do not use values obtained for the specific survey period and location in question, we cannot account for climate-related changes in mean decomposition time, thus hindering our ability to correctly estimate population trends.

Here, we used long-term data from the research site LuiKotale [65], Democratic Republic of the Congo (DRC), to investigate the impact of climatic conditions on nest decomposition. We investigated A) 15 years of daily rainfall and temperature data, and B) a total of 1,511 bonobo (*P*. *paniscus*) nests observed from construction to disappearance. As variables influencing nest decay time, we considered climate measured as 1) rainfall and 2) storms, as well as 3) habitat location (swamp versus dry forest), 4) construction type (height, position within tree, exposure), 5) construction behaviour, and 6) tree species.

1. Rainfall: as rain has been reported to be the most important factor affecting nest decay [38, 40, 41, 62], we expected high levels of precipitation to accelerate decomposition.
2. Storms: some authors found no correlation between average rain and decay time [58]. As rain can come in different forms [66], and thunderstorms with short and heavy rain may have a different influence on nest decay than continuous, lighter rain, we expected the number of storms, characterized by a high level of precipitation in a short time, to accelerate nest decay.
3. Habitat location: in contrast to heterogeneous primary forests on slopes and plateaus, those in riverine valleys and swamps are more protected from storms, due to their topographical position. Therefore, we expected nests built in heterogeneous dry forests to decompose faster than those built in swamps.
4. Construction type: if 1 or 2 were true, we would expect the effect to be more pronounced in nests built a) high rather than low in trees [40], and b) on side branches rather than in the fork at the treetops. This is because the wind forces bend upper trunk parts more than lower ones, and distal positions more than central ones. We also expected nests to decay faster when c) open to the sky, as they would be more exposed to the elements than those protected by upper layers of foliage [40]; and when d) formed by the integration of material of several trees [67], rather than of a single tree.
5. Construction behaviour: following recent findings [25], we expected nests constructed for rainy or colder nights to persist longer, as more foliage is used for thermal insulation and solid branches in single trees are chosen over smaller branches integrated from several trees to withstand strong winds accompanying changes in weather.
6. Tree species: as tree species rather than nest characteristics have been reported to influence decomposition time [38, 64, 68], we assessed differences between the most common trees used for nesting by the bonobos of LuiKotale.

We modelled the average time for a bonobo nest to fully decay in a Bayesian framework, using a gamma survival model describing the time between nest construction and full decomposition [69, 70]. We then compared our results with those obtained using the recommended application of estimating nest decay with a single revisit of a marked nest, using logistic regression [57], a time-efficient and retrospective method.

## Material and methods

### Study area

**Fig 1:**
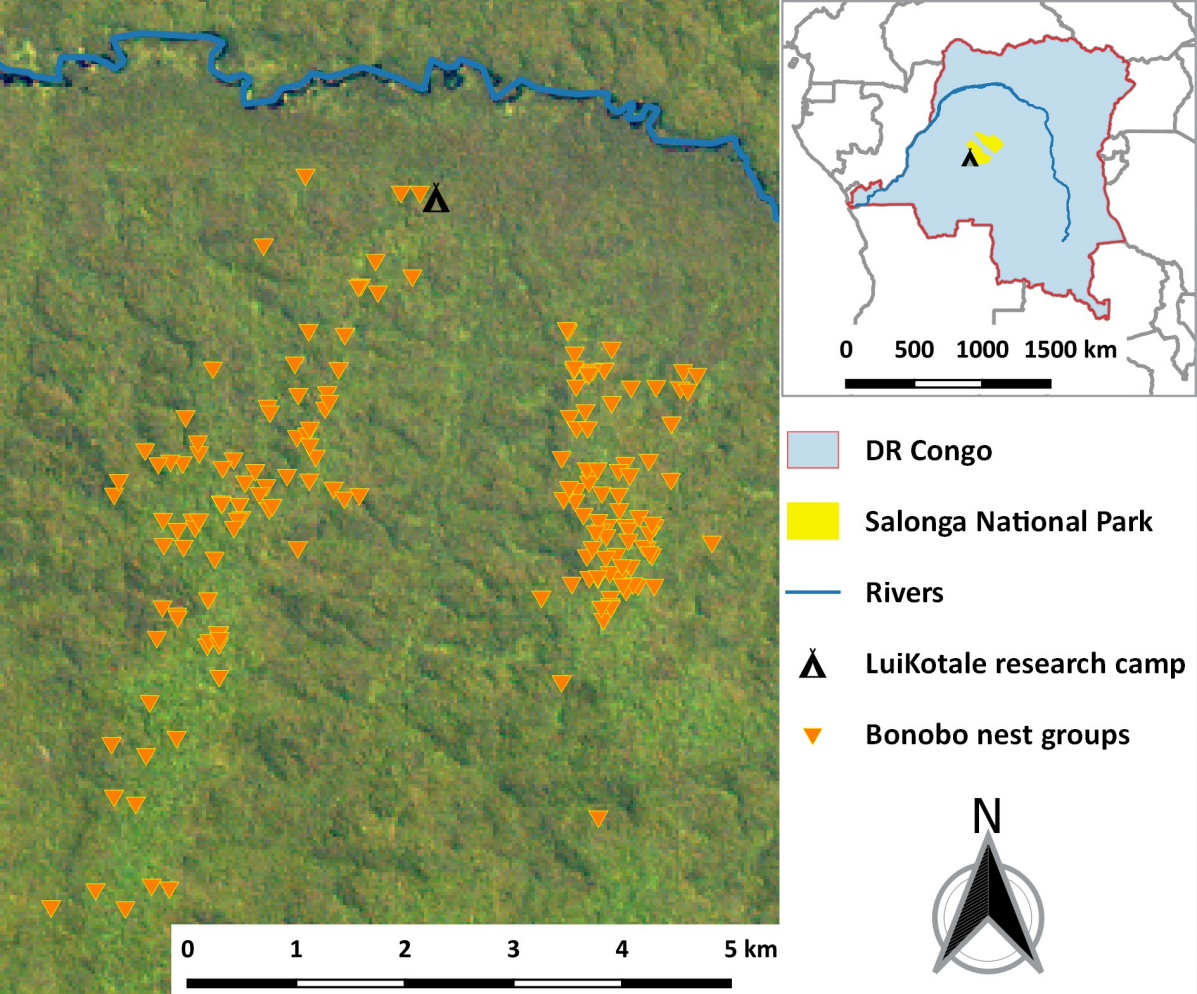
**Nest groups (n = 182; orange triangles) monitored in the LuiKotale Study area (2003–2018) situated west of Salonga National Park (yellow) in the Democratic Republic of the Congo (light blue).** Landsat-8 image courtesy of the U.S. Geological Survey.

We collected data between 2003 and 2018 at the research site LuiKotale (2°45.610′ S, 20°22.723′ E), west of Salonga National Park (SNP), DRC, a World Heritage Site of Nature, and a stronghold for wild bonobos [71] (Fig 1). The study site is situated in an area of lowland heterogeneous primary forest [65]. Here, two bonobo communities were habituated to human observers by 2007 (Bompusa West; ca. 40km^2^ home range) and 2015 (Bompusa East; ca. 30km^2^ home range), respectively [72]. The climate at LuiKotale is equatorial, with abundant rainfall throughout the year, except for a short dry season in February and a longer dry season between May and August.

### Data collection

#### Climate data

We measured daily rainfall (mm/m^2^) twice per day (at 6:00 and 18:00) using a rain gauge open to the sky. We measured minimum and maximum daily temperature (°C) using a thermometer in the forest. We recorded weather (i.e. rain, sun or clouds) in four time intervals: (a) 6:00–10:00; (b) 10:00–14:00; (c) 14:00–18:00; (d) 18:00–6:00 (i.e. night time); with interval (c) being the time during which bonobos built nests. This allowed us to extract the following variables: 1) daily rainfall throughout the lifetime of a nest; 2) daily number of storms throughout the lifetime of a nest, with storm being defined as a single interval of rain in an otherwise sunny day, with a minimum of 20 mm of rainfall within the time intervals mentioned above. The rain had to occur in the evening or night, as morning storms are extremely rare in the study area; 3) rain on construction date (i.e. if rain was recorded in interval (c), on the day of construction); 4) minimum temperature on construction date; and 5) differential temperature on construction date (max−min °C) to eliminate potential bias due to the use of different thermometers across time. We then compiled a full climatic database for the 15 years of study (2003–2018) to investigate 1) trends in climate conditions at LuiKotale and 2) differences between survey periods.

#### Nest decay data

We derived data on nests from direct follows of bonobos. Field staff marked the location of all nest sites included in this study in the evening, revisited them the morning after construction, and recorded GPS coordinates and habitat type. For each nest, we noted: 1) height (m); 2) exposure; 3) position in tree; and 4) construction type. For each nest tree, we recorded 5) height (m); 6) lowest branch (m); and 7) species (Table 1). We registered types of nest construction across two periods, nine years apart (Table 2). Period 1 (P1) included nests constructed across 35 months between August 2003 and July 2007. Period 2 (P2) included nests constructed across 24 months between July 2016 and June 2018. We further distinguished five surveys, lumping nests into ten to 13 consecutive months, reflecting a full year of data collection (Table 2): 1) August 2003–July 2004 (Survey 1, S1); 2) February 2005–December 2005 (Survey 2, S2); 3) July 2006–July 2007 (Survey 3, S3); 4) July 2016–June 2017 (Survey 4, S4); 5) July 2017–June 2018 (Survey 5, S5). S1 included the nests analysed by Mohneke and Fruth [73], which included 24 fresh nest groups (n = 218 individual nests) between August 2003 and February 2004 and applied SCNC and marked nest count methods.

**Table 1:** Nest type categories definitions, and sample sizes used in this study.

Factor	Description	Categories		Number of nests
Forest type (*F*)	Primary heterogeneous inundated forest	Swamp		84
	Primary heterogeneous forest on *terra firma*	*Terra firma*		1,427
Nest exposure (*E*)	Nest closed to sky by above vegetation	Close		544
	Nest open to sky	Open		967
Nest position (*P*)	Side branch	Side branch		1,326
	Treetop	Top		185
Nest construction type (*C*)	Nest located in a single tree	Single tree		1,257
	Nest using branches of 2 or more adjacent trees	Integrated		254
Nest absolute height (*A*)	Nest height from the ground (m)	Low	= <13	326
		Medium	13–23	874
		High	> = 23	311
Nest relative height (*H*)	Nest height from the ground (m) / Tree height (m)	Low	<0.8	234
		Medium	0.8–0.95	937
		High	>0.95	340
Average precipitation (*W*)	Average daily precipitation during a nest’s lifetime (mm)	Low	<3	210
		Medium	3–9	1,056
		High	>9	245
Average storms (*S*)	Average daily number of storms during a nest’s lifetime	Low	<0.02	196
		Medium	0.02–0.12	1,059
		High	>0.12	256
Minimum temperature (*T*)	Minimum temperature recorded on day of construction (°C)	Low	= <18	261
		Medium	18–22	1,084
		High	> = 22	166
Differential temperature (*D*)	Difference between maximum and minimum temperature at day of construction (°C)	Low	= <4	302
		Medium	4–8	868
		High	> = 8	341
Rain at construction (*R*)	No rain between 14:00 and 18:00 on day of construction	No		1,291
	Rain between 14:00 and 18:00 on day of construction	Yes		220
Tree species (*Sp*)	Tree species where nest was constructed. The 10 most recurrent species were assigned to a specific category. All other species were included in the eleventh category (including integrated nests).	1–11		See S1 Table

**Table 2:** Period- and survey-specific information.

Period	Survey	Study length (dates)	Number of nest groups	Number of individual nests	Number of censored individual nests (%)
P1	S1	12 months (08/2003–07/2004)	33	278	45 (16.2%)
	S2	10 months (02/2005–12/2005)	30	305	23 (7.5%)
	S3	13 months (07/2006–07/2007)	23	249	22 (8.8%)
Total (P1)		35 months (08/2003–07/2007)	86	832	90 (10.8%)
P2	S4	12 months (07/2016–06/2017)	47	450	26 (5.7%)
	S5	12 months (07/2017–06/2018)	39	329	87 (26.4%)
Total (P2)		24 months (07/2016–06/2018)	96	679	113 (16.6%)
Total (study)		59 months	182	1,511	203 (13.4%)

Although one person only (LB) estimated and followed the majority of nests, we divided nest height by tree height to use relative nest height accounting for possible differences in observer estimates. All relevant field research staff were trained in estimating nest and tree height using a clinometer. We visited nests on a weekly basis, recording age class, until complete decomposition. We defined five age classes [32] distinguishing nests consisting of 1) fresh and green leaves; 2) green but dry leaves; 3) brown/black leaves; 4) no leaves but the structure of broken and bent branches still visible; and 5) nests recognizable only if the place of construction was known to the observer. We considered a nest fully decomposed when it entered class 5. We calculated nest “age” *post hoc* as the number of days between the day of construction and the day before it was found to be fully decomposed. If a nest was not fully decomposed at the end of the study, we included it as “censored” [74]. Consequently, in censored nests, the time to decay was smaller than the true but unknown time to decay.

This study was purely observational, involving nests left behind by bonobos. The methods described above complied with the requirements and guidelines of the ‘Institut Congolais pour la Conservation de la Nature’ and adhered to the legal requirements of the host country, DRC.

### Statistical analysis

#### Defining factors

The following nest characteristics were binary: forest type (*F*), nest exposure (*E*), nest position (*P*), nest construction type (*C*) and rain at construction (*R*). As nests were constructed in 85 different tree species (*Sp*), we assigned a single category to the most recurrent 10 tree species (1 to 10), and included all other species in a separate category (i.e. 11) (S1 Table). Because the top 10 tree species varied between P1 and P2, we repeated this process for 1) all investigated nests; 2) nests constructed in P1; and 3) nests constructed in P2.

The following variables were continuous: nest height (*A*), relative nest height (*H*), average precipitation (*W*) and daily number of storms (*S*). We classified these into the following three categories based on their mean and standard deviation (SD): “Low” (mean– 1 SD), “High” (mean + 1 SD) and “Medium” (anything in between) (Table 1).

Some of the factors described in Table 1 measured the same phenomenon in different ways (i.e. absolute nest height / relative nest height; minimum temperature / differential temperature). To avoid problems of overfitting [75], we fitted four different models including all possible combinations of these variables and selected the top ranked models by using Pareto-smoothed importance sampling (PSIS) [76]. Average daily rainfall and average number of storms throughout the lifetime of a nest posed a similar issue, as we expected these two variables to be correlated (if there is a lot of rain, there are many storms). However, the phenomena they described were different and could both be important in driving nest decomposition. Therefore, we first fitted a model including both variables, and verified that the posterior distribution of the parameters was not correlated (i.e. no overfitting) following Vehtari *et al*. [76]. Then, we looked at the decrease in expected log predictive density (ELPD) given by removing one or the other covariate [77]. A big decrease would suggest that the removed covariate exerted a high contribution to the predictive power of the model. A small decrease would suggest a low contribution. We applied the same process to all other variables, running different models and leaving one variable out at a time. By looking at the decrease in ELPD values given by removing a particular variable, we evaluated the importance of each factor in driving nest decomposition time. We performed model comparison and selection using the R package “loo” [76].

#### Modelling nest survival

Nest survival analyses have been previously conducted using four main methods: Kaplan-Meier models [61, 73], survival analyses [40] provided by the software MARK [78], Hidden Markov chain analysis [58, 79] and logistic regressions [38, 57]. Here, we modelled the average time to full decay of a bonobo nest using a Bayesian gamma survival model describing the time between nest construction and full decomposition [70]. The gamma distribution properly describes the length of time between events and provides more flexibility than the typically used exponential distribution [69]. In order to include censored nests (i.e. nests for which the exact time to full decay was not observed), we modelled fully decayed nests using a gamma cumulative probability distribution, which provided the probability that a nest has disappeared after a certain number of days. Conversely, we modelled censored nests by using the complementary cumulative probability distribution, which essentially returns the probability a nest has not disappeared after a certain number of days [70].

In mathematical terms, for each fully decayed nest *i*, we modelled the days to full decay *Dd* as

Dd∼gamma(k,θ) Eq 1

And censored nests *Dc* as

Dc∼1−gamma(k,θ) Eq 2

where the shape parameter of the gamma distribution is *k*, defined as the mean decomposition time μ multiplied by the rate parameter θ

k=μ*θ

The mean decomposition time μ for each nest *i* can be modelled depending on nest-specific factors, using a linear model with a log-link function, such as

$$\mu_{i}=exp\;({ϝ}_{j}[F_{i}]+\varepsilon_{j}[E_{i}]+\pi_{j}[P_{i}]+\gamma_{j}[C_{i}]+\alpha_{j}[A_{i}]+\eta_{j}[H_{i}]+\omega_{j}[W_{i}]+\sigma_{j}[S_{i}]+\tau_{j}[T_{i}]+\delta_{j}[D_{i}]+\rho_{j}[R_{i}]+\psi_{j}[{Sp}_{i}])$$ Eq 3

where Greek letters represent specific parameters of variables estimated by the model for each category *j*, and capital letters represent variables (see Table 1). This allowed us to investigate differences in mean decomposition time for nests belonging to each category within our variables. We assigned a weakly informative prior to each parameter *y* in the linear model

$$y_{n}∼normal\;(0,5)$$ Eq 4

where *y* represents any of the parameters *n* in Eq 4, and a positive weakly informative prior to the parameter θ in Eqs 1, 2 and 3

θ∼gamma(0.1,0.1) Eq 5

Finally, in order to validate our model, we reanalyzed an already published dataset [73].

Another method used in great ape conservation is logistic regression analysis [57]. This method has the advantage of allowing average nest decay time to be estimated retrospectively, with only one revisit of marked nests. It has thus been recommended as the most cost-effective approach for obtaining site-specific nest decay time [34, 35]. Published studies using logistic regression analysis have focused on African great apes (S2 Table), whilst orangutan researchers have mainly used the Hidden Markov Chain method [58] (but see [80, 81]). Logistic regression studies differed considerably in terms of the number of nests included, survey length and the time between the day the nests were marked and the day they were revisited (S2 Table). However, nest decay is not constant across time [41] and nests built at the same time could decay at different rates [40]. Therefore, if the sample of nests is of inadequate size, or not representative of the climatic variation across the period of study, average nest decay estimates can be biased [57]. In order to assess the reliability of this method, we used logistic regression to estimate mean decomposition time of the same published dataset used to validate our model [73], following the protocol described by Kouakou *et al*. [38]. We investigated 1) different survey lengths: a) full length of study), and b) half-length of study; and 2) different intervals between nest marking and revisit: a) 2 weeks, b) 1 month, c) 3 months after the last nest group was marked, d) 3 months after each nest group was marked [64], and e) a random number of days (between 7 and 360) after the nest was marked [82]. We then binary labelled the individual nests as “Decayed” = 0, if the date of revisit occurred after the known date of decay, or “Alive” = 1, if the date of revisit occurred before the known date of decay. We compared the results to the known mean decay time [73] and adopted the most reliable scenario to investigate the effect of sample size on the precision of the estimate. Here, we randomly selected a sample of nests comprising a) 75%, b) 50% and c) 25% of the nest groups included in the original dataset (10 random draws each) and compared the results with those obtained by 1) Mohneke and Fruth [73] and 2) our gamma survival model. In addition, to further explore the effects of sample size on estimated decay time, we repeated the same analyses for a larger dataset: S4, presented in this study.

We developed our gamma survival model (S1 Appendix) in ‘Stan’, a state-of-the-art platform for Bayesian statistical modelling [83], using the R interface RStan [84]. We used R Version 4.0.2 [85] to run two chains for each survival model (4,000 iterations; 2,000 of warmup), to perform exploratory and logistic regression analyses and to create figures.

## Results

### Climate

Between 2003 and 2018, the climate recorded in LuiKotale showed a significant trend of decreasing average rainfall (linear regression: *R*^*2*^ = 0.363; *p* = 0.013) and differential temperature (*R*^*2*^ = 0.248; *p* = 0.049), but revealed no difference in the number of storms per year (S1 Fig). Consequently, in 2003−2007 (P1), average daily rainfall in the lifetime of a nest was higher than in 2016−2018 (P2) (Wilcoxon test: *W* = 279021, *p* < 0.001). P1 also showed larger temperature variation between day and night on construction date (*W* = 257941, *p* < 0.001). However, the daily number of storms in the lifetime of a nest was higher in 2016−2018 than in 2003−2007 (*W* = 195465, *p* = 0.002).

**Fig 2:**
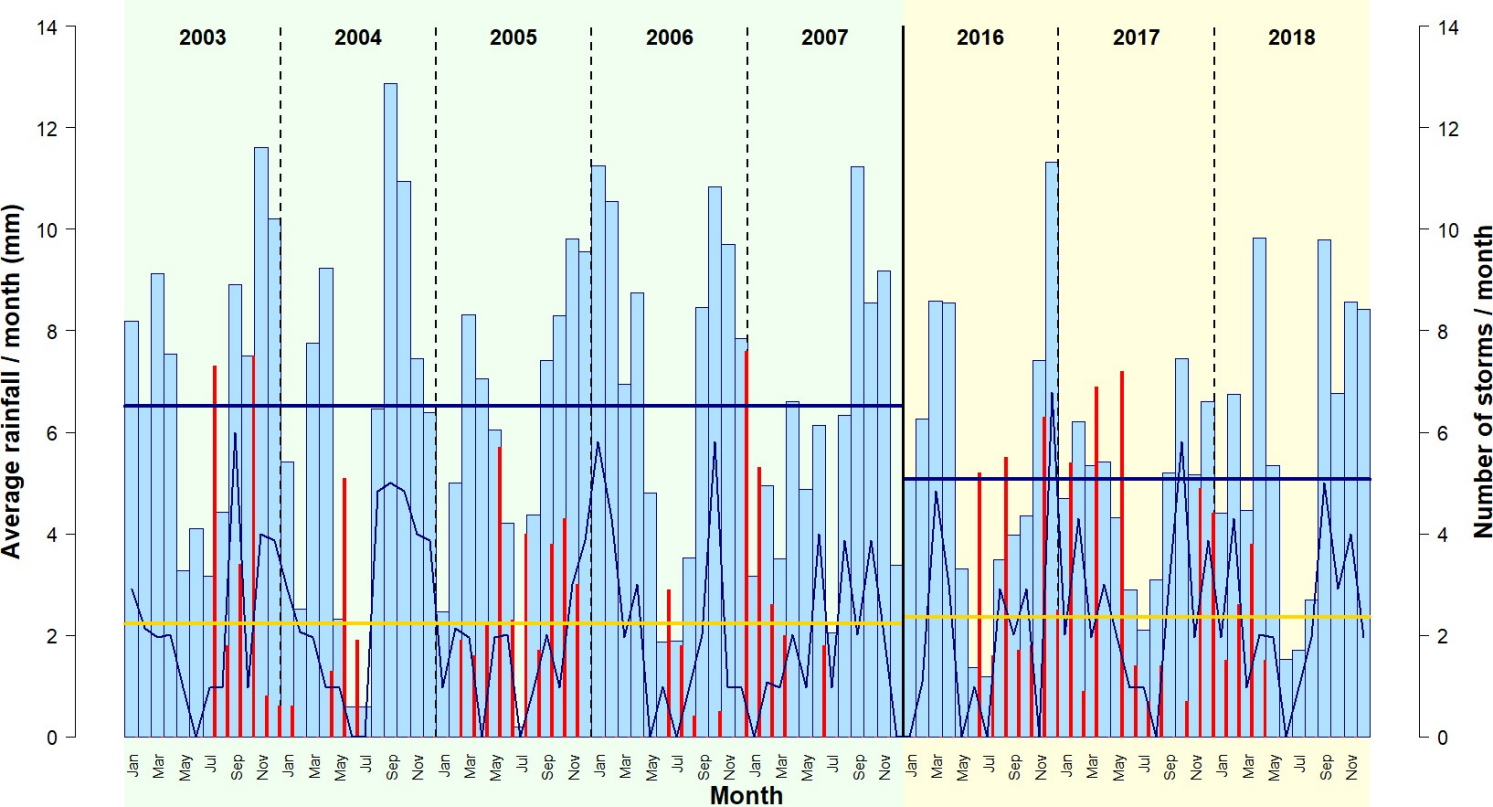
**Monthly trend of average rain (light blue bars) and number of storms (dark blue line) across 1) periods (P1 = green background; P2 = yellow background), and 2) years (delimited by vertical lines).** Horizontal solid lines represent 1) average monthly rainfall between periods (blue) and 2) average monthly number of storms between periods (yellow). Red vertical bars show the number of nests included in the study in each month (divided by 10, for graphical purposes).

These results were also confirmed by the analysis of monthly climate data across our two study periods (Fig 2). When investigating average monthly rainfall and differential temperature, a marked decrease from P1 to P2 became evident; despite a tendency for more storms in P2, the number of storms per month was statistically similar across periods (S2 Fig). Accordingly, 2003−2007 was a wetter period, with larger day/night temperature variation.

### Model diagnostic and validation

Following the results of the model selection procedure (S2 Appendix), we included both average rainfall (*W*) and daily number of storms (*S*), defining the mean nest decomposition time μ for nest *i* by modifying Eq 3 as follows,

$$\mu_{i}=exp({ϝ}_{j}[F_{i}]+\varepsilon_{j}[E_{i}]+\pi_{j}[P_{i}]+\gamma_{j}[C_{i}]+\eta_{j}[H_{i}]+\omega_{j}[W_{i}]\sigma_{j}[S_{i}]+\delta_{j}[D_{i}]+\rho_{j}[R_{i}]+\psi_{j}[{Sp}_{i}])$$ Eq 6

where Greek letters represent specific parameters of variables estimated by the model for each category *j*, and capital letters represent variables (see Table 1). All models converged well (S3 Fig), with potential scale reduction factor, “Rhat” [76], being equal to 1 for all parameters. To assess the reliability of our estimates, we examined the Pareto *k* diagnostic [76], showing that our model was well specified (S3 Table) and results were credible (S4 Fig).

Finally, in order to validate the model accuracy, we reanalyzed the data from 2003−2004 published by Mohneke and Fruth [73], for which average decomposition time was reported to be 75.5 days (n = 218 nests, 95% confidence interval (95% CI) = 68.4−82.5). Our model returned an average decomposition time of 77.5 days (95% CI = 71.3−84.2), fully within the confidence interval reported previously.

### Nest decay time

We monitored 182 nest groups, totaling 1,511 nests across two periods (P1 and P2), 9 years apart. Monitored nests were constructed both in the dry (n = 643) and rainy season (n = 868), with all months being represented (average number of nests/month = 128; min = 50 (May), max = 180 (June)). Period- and survey-specific information are provided in Table 2.

**Fig 3:**
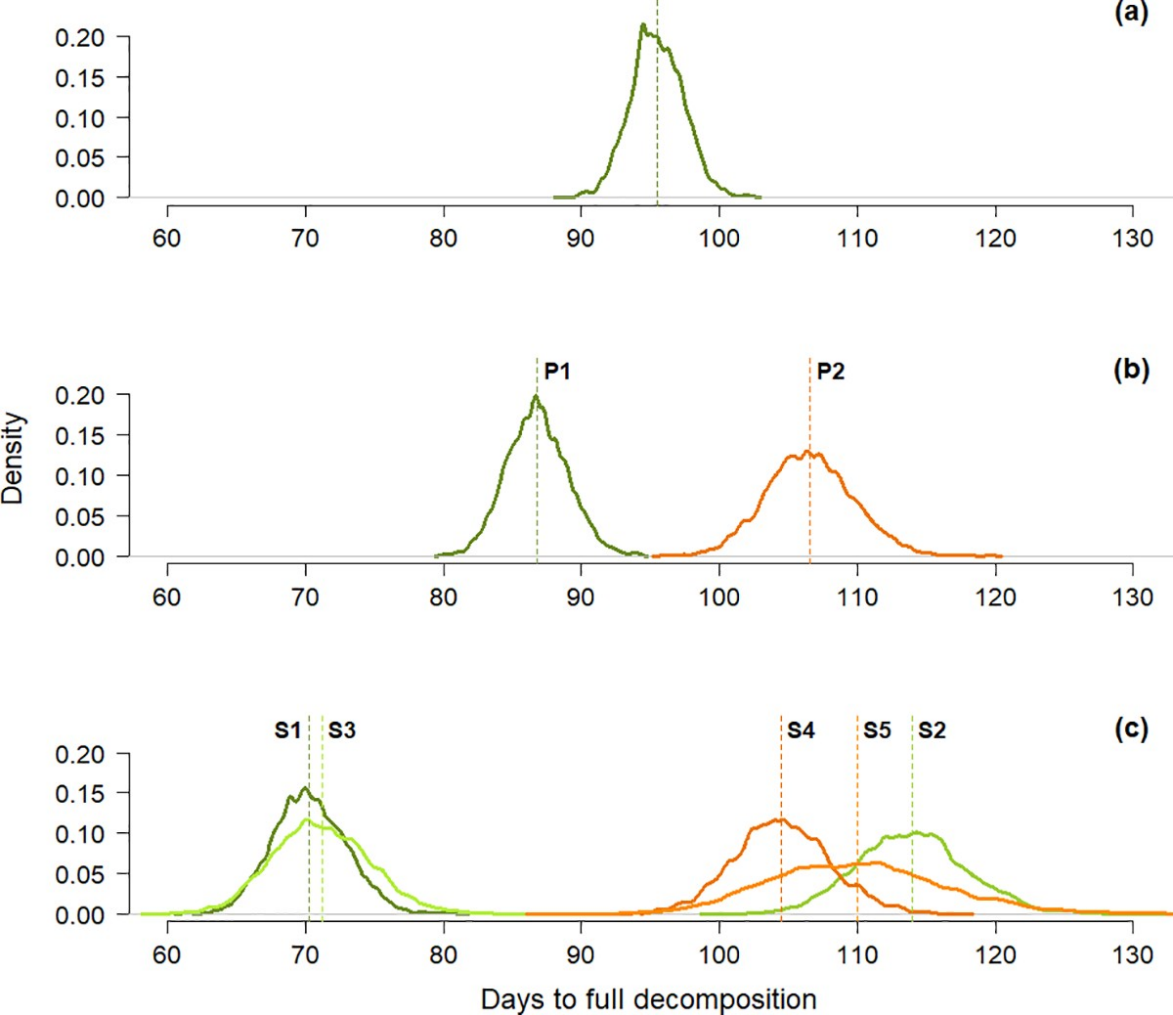
Posterior distribution of estimated mean decomposition time. a) All nests (n = 1,511); b) nests from the first (P1; n = 832; green) and second period (P2; n = 679; orange; c) nests from different surveys from P1 (S1 (n = 278); S2 (n = 305); S3 (n = 249); green curves) and P2 (S4 (n = 450); S5 (n = 229); orange curves). Coloured dashed lines show the mean values of the posterior distributions. Curves with larger breadth indicate higher uncertainty.

The estimated average nest decomposition time for the full dataset was 95.5 days (SD = 1.93, see Fig 3A). When looking at differences between periods (i.e. 2003−2007 versus 2016−2018), we observed a significant increase, with an average nest decomposition time of 87.5 days (SD = 2.22) in P1 and 106.7 days (SD = 3.12) in P2 (Fig 3B). These results were only partially supported by the analysis of yearly nest decomposition cycles (i.e. specific surveys), with S1 and S3 in P1 showing average decomposition times consistently shorter than all other periods. Interestingly, S2, including 305 nests from February–December 2005, showed a mean decomposition time similar to those recorded in P2 (Fig 3C).

**Fig 4:**
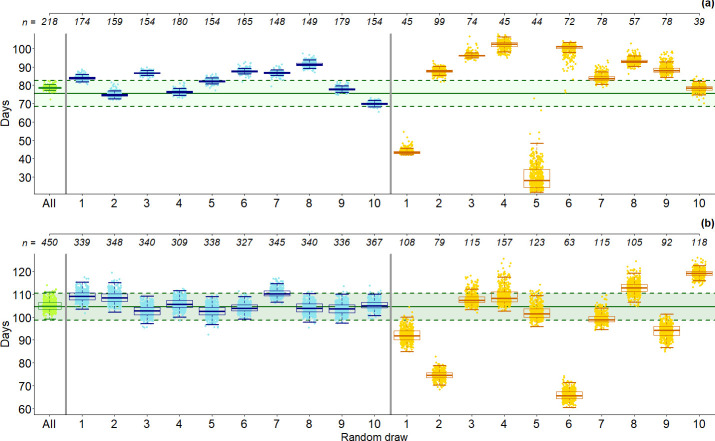
Estimated mean decay time of 10 datasets randomly selected from (a) Mohneke and Fruth [73] and (b) S4 [this study], using logistic regression [57]. Clouds of dots represent 500 mean decay times estimated via bootstrapping [38], with boxplots representing quartiles and variability of the bootstrap samples. From left to right: 1) full datasets (green) i.e. “All”; 10 random draws of 2) 75% of nest groups (blue); 3) 25% of nest groups (yellow). The upper x-axis shows the number of individual nests *n* in each draw. Green, horizontal lines show the reference decay value (mean, solid line) and 95% confidence intervals (dashed lines) for a) decay time for Mohneke and Fruth [73]; and b) S4 estimated via gamma survival model [this study].

Using the dataset analyzed in Mohneke and Fruth [73], we also conducted a logistic regression analysis. A randomly selected time to revisit (specific to each nest group) returned an average decomposition time of 78.2 days (95% CI = 77.3−80.6) (S4 Table), which we used to analyze datasets with reduced sample size. A reduction to 75% of the original dataset had limited effect on the estimated decay time (Fig 4). In contrast, when reducing sample size to 25% of the original dataset, particularly for S4 of our study (Fig 4), results became variable. A similar trend was observed when analyzing the reduced dataset using our gamma survival model (S5 Fig). However, here the estimated decay times were less variable than those obtained with a logistic regression analysis.

Finally, the time interval between nest marking and revisit influenced the estimated decay time when analyzing the data from Mohneke and Fruth [73] (n = 218), whereas results were more consistent for S4 (n = 450) (S4 Table).

### Factors affecting decomposition time

Table 3 shows that bonobo nests tended to persist longer if constructed (*C*) with material from single rather than multiple trees (i.e. integrated) and, in contrast to our expectation, if exposed to the sky (*E*).

**Table 3:** Factors influencing nest decomposition time: Results of the survival model.

Factor	Category	Parameter	Parameter mean (95% CI) Log-scale	Average decomposition time (95% CI) Natural scale (Days)
Forest type (*F*)	Swamp	ϝ_1_	0.52 (-6.13−7.48)	87.09 (75.00−100.01)
	*Terra firma*	ϝ_2_	0.62 (-6.08−7.59)	95.96 (92.14−99.91)
Nest exposure (*E*)	Close	ε_1_	0.42 (-6.13−6.94)	87.39 (82.20−92.61)
	Open	ε_2_	0.56 (-6.01−7.06)	100.18 (95.52−104.99)
Nest position (*P*)	Side branch	π_1_	0.52 (-6.16−7.02)	93.85 (89.95−97.84)
	Top	π_2_	0.65 (-6.04−7.14)	107.11 (96.35−118.56)
Nest construction type (*C*)	Single tree	γ_1_	0.67 (-5.53−6.89)	98.45 (94.41−102.62)
	Integrated	γ_2_	0.50 (-5.69−6.74)	82.95 (75.61−90.60)
Nest relative height (*H*)	Low	η_1_	0.43 (-5.02−5.79)	102.38 (93.05−112.04)
	Medium	η_2_	0.37 (-5.05−5.74)	96.43 (91.98−100.97)
	High	η_3_	0.29 (-5.13−5.61)	88.52 (81.31−96.06)
Average precipitation (*W*)	Low	ω_1_	0.30 (-5.20−5.83)	92.41 (80.64−104.89)
	Medium	ω_2_	0.35 (-5.17−5.88)	96.98 (92.81−101.21)
	High	ω_3_	0.26 (-5.23−5.80)	89.08 (80.09−98.98)
Average storms (*S*)	Low	σ_1_	-0.17 (-5.49−5.05)	46.05 (39.91−52.60)
	Medium	σ_2_	0.70 (-4.64−5.88)	109.41 (104.56−114.37)
	High	σ_3_	0.27 (-5.04−5.46)	71.82 (64.45−79.59)
Differential temperature (*D*)	Low	δ_1_	0.32 (-5.18−5.87)	93.13 (85.82−100.55)
	Medium	δ_2_	0.36 (-5.16−5.92)	96.70 (92.11−101.24)
	High	δ_3_	0.33 (-5.17−5.88)	94.17 (87.40−101.51)
Rain at construction (*R*)	No	ρ_1_	0.61 (-5.91−7.32)	95.23 (91.36−99.17)
	Yes	ρ_2_	0.62 (-5.89−7.34)	96.86 (88.48−105.49)
Scale parameter		θ	0.02 (0.02–0.02)	*NA*

For each category (parameter) included in the model we show 1) Parameter mean: posterior mean with 95% confidence interval (95% CI) (log-scale); and 2) Average decomposition time: with 95% confidence interval (95% CI) (natural scale (Days)).

Bonobo nests also survived longer if built in the very top of a tree crown (*P*) (in contrast to side branches) and at lower heights (*HT*) (in contrast to nests built in upper parts of a tree). However, nests built on side branches in the apical section of a tree had a decomposition time 29 days shorter than those built in the fork at the top of the tree (i.e. top nests). As a result, nest height (*HT*) did not turn out to be a significant predictor of average nest decomposition time (Table 3; S6 Fig and S2 Appendix). Forest type (*F*) had limited influence on nest decomposition time (S2 Appendix). In contrast, our results suggest that tree species (*Sp*) was an important factor (S2 Appendix), with nests constructed on species such as *Scorodophloeus zenkeri* showing a shorter decomposition time (average = 89.93; 95% CI = 74.16−107.55) than nests built on more solid species, such as *Anonidium mannii* (average = 109.82; 95% CI = 87.99−133.28). S1 Table shows the estimated nest decay time for the most recurrent tree species.

**Fig 5:**
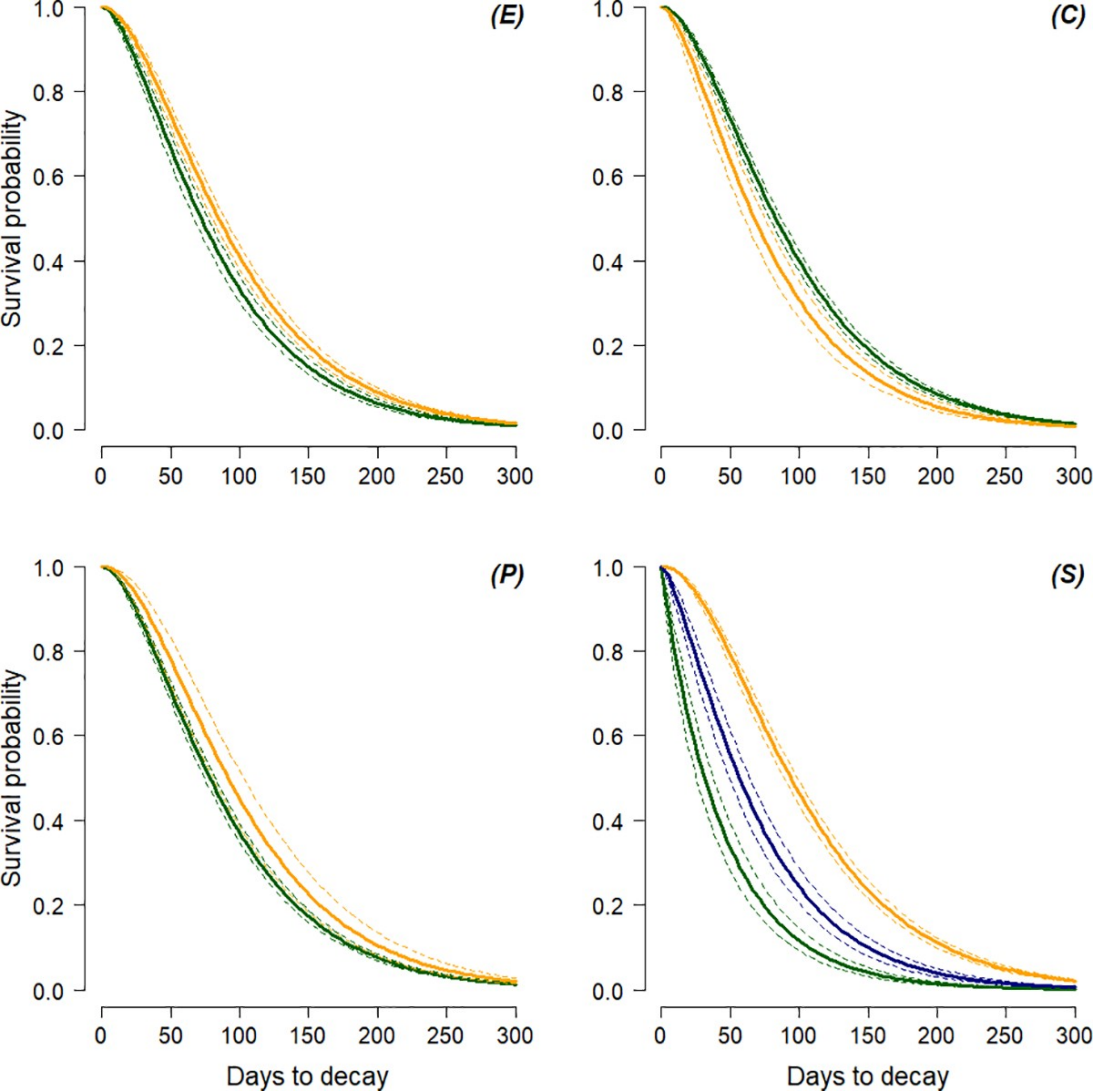
Factors influencing nest survival. Survival curves (bold, coloured lines) and 95% confidence intervals of the mean (dotted, coloured lines) for: (E) Exposure of nest: “Open” (orange) and “Closed” (green); (C) Construction type: “Integrated” (orange) and “Single tree” (green); (P) Position: “Side branch” (green) and”Top” (orange); (S) Nest exposed to number of storms: “Low” (green),”Medium” (orange) and “High” (blue).

As expected, nests exposed to high levels of precipitation across their lifetime (*W*) showed the fastest decomposition (Fig 5). However, there were inconsistencies across periods regarding the conditions “intermediate” and “few” rains, with nests exposed to “few” lasting the longest in P1, and nests exposed to “intermediate” surviving the longest in P2 (S5 Table). Conversely, the number of storms in the lifetime of a nest (*S*) was consistent across periods. In contrast with our prediction, nests exposed to fewer storms survived less than those exposed to numerous storms, with the longest decomposition time being exhibited by nests exposed to intermediate ones (Fig 5). Here, nest construction type differed significantly between the different categories. Among those exposed to a “Low” number of storms, the proportion of integrated nests was almost twice that observed for nests exposed to a “High” (Chi-squared test: *X*^*2*^ = 5.2, *p* = 0.022) or “Medium” number of storms (*X*^*2*^ = 15.2, *p* < 0.001). Conversely, the proportion of top nests was four times higher among the nests exposed to a “High” number of storms (*X*^*2*^ = 17.3, *p* < 0.001). Finally, neither rainy condition (*R*) and/or differential temperature between day and night (*D*) at day of construction had a significant influence on nest decomposition time (S6 Fig and S2 Appendix).

## Discussion

Long-term studies have revealed the dramatic impact of climate change on Central African rainforests. Only recently, an analysis of phenological data covering a 32-year period raised severe concerns of the impact of climate change on Central African forests [10]. Our study, although only covering 15 years of climate data, reaffirms that climate change is impacting upon the very heart of the second largest forest area on our planet, the Congo Basin. Here, we showed the steady decline of rainfall across years, and the impact of this on bonobo nest decay times. This connection is relevant to all great ape density estimations using distance sampling, and is therefore of major importance to the interpretation of all biomonitoring estimates, past, present and future, inferring global significance in the conservation efforts of great apes in the wild.

Many studies have reported an effect of climate on great ape nest decomposition time, with drier conditions resulting in longer decay times [38, 40, 64, 86]. Our study links the decomposition of 1,511 bonobo nests to climatic data spanning a 15-year period, and supports this claim. In LuiKotale, bonobo nests constructed in 2016–2018 showed a mean decay time 17 days longer than nests constructed in 2003–2007, when average monthly rainfall was higher (Fig 2). We also found that average nest decay time varied significantly from one year to another in the same period, with nests in S2 exhibiting a decomposition time one month longer than those constructed in the previous (S1) and subsequent (S3) years. The year 2005 (S2) was characterized by a very short dry season, with a single month (July) of extremely limited precipitation. In addition, most rain and storms in that year were concentrated at the end of the survey (between November and December 2005) (Fig 2). Therefore, a long dry climate at the beginning of the survey (February–August) may have triggered a longer decay time for most nests. Indeed, nests constructed between February and August 2005 showed an average decay time 20 days longer than those constructed after the end of the dry seasons, highlighting the atypical conditions of that year. This clearly demonstrates how using a decay time estimated in a different period, or in a different survey (e.g. S2), could result in a severe bias in bonobo density estimates.

### Factors affecting decomposition time

Several studies have reported the amount of rain occurring in the lifetime of a nest as the most important factor affecting nest decomposition time [38, 40, 64]. However, others did not find a clear pattern in support of this claim [58]. Here, we assumed events of extreme precipitation (i.e. storms) to affect nest decomposition time more than continuous rains. By investigating both factors, we found that the occurrence of storms, rather than rainfall, was the most important parameter explaining differences in nest decay time (Table 3, Fig 5 and S2 Appendix). As expected, high values of rain were related to faster decomposition time (Table 3). However, the correlation was not significant (S6 Fig), and the observed pattern varied between periods, with P1 showing no differences between rain categories, and P2 showing nests experiencing a “Medium” amount of rain lasting longer than nests experiencing both “Low” and “High” amounts of rain (Table 3 and S5 Table). In contrast, the number of storms showed a consistent, yet unexpected pattern. In line with our expectations, “High” numbers of storms triggered a decomposition time 24 days faster than the mean value of 95 days. However, “Low” numbers of storms were associated with a decomposition 49 days faster (Table 3). With 88% of nests experiencing “Low” numbers of storms being constructed in the dry season, either in February, or between May and August, our results suggest a shorter decay time for nests constructed in the dry season, at odds with the expectation of a longer decay time. We suspect that the reasons for such a pattern are behavioural. It has been recently suggested that chimpanzees adjust nest construction type in response to the weather [25]. Chimpanzees built thicker, more insulated nests in colder conditions, increasing the number of broken branches and choosing larger support branches, in moister and windier weather [25]. Here, we found no influence on decay time of rain and temperature at the time of nest construction, the proxies we chose to investigate a behavioural influence on nest decomposition time (Tables 3 and S6 Fig). In addition, we did not record nest construction measures, such as those described in [25, 40, 87]. However, we found nests experiencing a “Low” number of storms to be less frequently constructed in the treetop, and more frequently comprising materials from more than one tree (i.e. integrated nests). Indeed, our results showed that integrated nests lasted 15.5 days fewer than those constructed using material from a single tree, whilst a nest in the treetop exhibited a longer decomposition time than one built on side branches (Table 3). These results suggest that bonobos in LuiKotale exhibit flexible nest building behaviour, constructing less durable (e.g. located on side branches) but more comfortable (e.g. integrated) nests during the dry season when strong nest support is not required because of a predictable absence of storms. Although involving significantly fewer rains, we observed a similar number of storms per month in 2016–2018 (P2) as in P1 (2003–2007) and a less obvious dry season (Fig 2 and S2 Fig). This suggests that, in P2, rain was less predictable and was more likely to appear during a storm. Bonobos might have adapted to such a climate by strengthening nest structure to cope with unpredictable and intense precipitation, thus enabling longer decay time.

Other studies found nests exposed to the open sky to decay faster than those protected by upper foliage [40]. However, we found an opposite trend, with “Open” nests surviving longer than “Closed” nests (Table 3 and Fig 5). The reason for this may also be behavioural. Building a nest open to the sky allows an ape to dry quicker, avoiding exposure to persistent dripping water from above foliage after rain [88]. Such open nests may require stronger support and a thicker structure to resist wind [25], potentially resulting in open nests lasting longer than those shielded by vegetation but not built as strong. In addition, and in contrast to our expectations, the difference between the height of the nest and its survival was not statistically significant, further supporting the importance of bonobo nest-building behaviour on decay time.

In order to better understand the adaptability of great ape nesting behaviour to climatic conditions, future studies should record nest structure in greater detail. In particular, such detail includes measures of nest thickness and strength [25, 26, 87], material used [31, 40] and nest position on the branches (i.e. distance from the trunk).

Here, we defined a storm in a rather crude way, looking at single, abundant bursts of rain (minimum of 20 mm/m^^2^^), following bright sunshine in the afternoon or clear sky at night. However, a more sophisticated classification would incorporate wind speed [25]. Indeed, it is likely that strong winds, in combination with heavy rain, are very effective in dismantling great ape nests, particularly those constructed further away from the tree trunk or with flimsier supports.

Habitat (i.e. forest type) did not significantly affect decomposition time in LuiKotale (S6 Fig), probably because of the limited number of nests in our study constructed in swamp forest (5%). However, in accordance with other studies [38, 64, 68], we found that the tree species had an important influence on nest decomposition time (S2 Appendix). Here, we observed a large variation between and within species (S1 Table). *Dialium* species were the most frequently used, with “Maku pembe” (comprising 5 species) being used in 25% of nests (S1 Table). However, *Dialium* is the most frequently occurring genus in LuiKotale [89]. We also found that nests constructed in species such as *Anonidium mannii* (129 days) or *Plagiostyles africana* (113 days) lasted considerably longer than those constructed in trees such as *Scorodophloeus zenkeri* (84 days) or *Trichoscypha arborescens* (77 days; only used in P2) (S1 Table). To further investigate this phenomenon, future studies should include information on the phenotypes and biomechanical properties of the nesting tree species [26, 87].

### Nest decay and great ape conservation

Nest decay values are widely used in the monitoring of great ape populations to convert nest density into ape density [35] and are therefore of great importance when assessing IUCN extinction risk categories. In recent years, new protected areas have been created following nest count studies and great ape conservation strategies, such as Moyen-Bafing National Park, Guinea [90]. Management plans and conservation strategies continue to be based on nest count surveys, as are studies assessing the effectiveness of conservation interventions [34, 91].

Many studies have shown that the average nest decomposition time is extremely variable [39, 58], thus recommending the use of values reflecting time- and site-specific nest decomposition [35, 57]. However, until 2008, the few published decay times were commonly applied to all great ape surveys [40]; subsequently, best practice guidelines for great ape monitoring were published, discouraging such decisions [35]. In recent years, some studies have included survey-specific decay time by either observation [48, 79, 80, 82] or modelling [38, 53, 54, 81, 92–95]. Others have incorporated values obtained in the same area from an earlier survey [51, 55, 96–99], from a site close to the surveyed area [34, 50, 58, 60, 99, 100], or from averaged published values [101]. Our findings highlight the problematic nature of this approach. Even within a short period of 3 years (P1 in this study), decay time showed a between-years fluctuation of as many as 44 days (Fig 3). As an example, if a bonobo SCNC survey was performed at LuiKotale in 2005, in which a constant nest production rate (= 1.37 nest/individual [35]) and decay time calculated from the year before (i.e. S1) were applied, a real bonobo population of 30 individuals would be overestimated by 60%, to 48 individuals. In addition, our results suggest that it is problematic to model nest decay using tree species only [68], or abiotic factors such as rainfall and habitat type [53]. Both abiotic and biotic factors must be included in order to obtain reliable estimates, reflecting the high variability observed in our study.

The most reliable estimates of nest decomposition time are obtained via continuous monitoring of a sample of nests large enough to be representative of the period of survey [35]. However, such a protocol is infeasible in most cases, where financial and time resources are limited. Therefore, more time-efficient methods, such as the retrospective estimation of nest decay with a single revisit of a marked nest [57], are recommended [34]. Using a logistic regression on subsets of our long-term data, we obtained consistent estimates in many cases (Fig 4 and S4 Table). However, we also found a considerable amount of variation, mainly due to 1) unrepresentative sample size and 2) inappropriate interval between nest marking and revisit.

When we mimicked a smaller sample size by randomly reducing our full dataset to 75 and 25% to investigate decay representativity, biased decay time estimates became more apparent the smaller the sample (Fig 4 and S5 Fig). This effect was more pronounced for the 1-year (S4; July 2016–June 2017), than for the 6-month survey [73]. In field conditions, this happens when nest groups included in the decay study do not represent the possible factors affecting decay [41]. According to our results, the time between nest marking and revisit also affected the precision of decay time estimates. Here, inconsistent estimates became apparent when reanalyzing the survey by Mohneke and Fruth [73], while the analysis of S4 returned consistent estimates, possibly because of the larger sample size. This was more pronounced when evaluating different times of revisit for half the survey time (i.e. 3 months in [73]; 6 months in S4) (S4 Table). Great ape nests do not decay steadily [41], but short periods of heavy rains, for example, can accelerate decomposition of many nests at a time. Therefore, including or excluding such periods can affect the precision of the estimates, particularly for low sample sizes. In addition, when we set the revisit time at 3 months after the last nest group was marked, in the analysis of the dataset from Mohneke and Fruth [73], all nests (except one) were already decayed at revisit, rendering the method invalid. It is thus important to select a revisit time that corresponds to the known or expected decay time for the area of study. While too few days can exclude or include periods of fast nest decay, excessively long times can result in no nests being “alive” at revisit, thus making analysis impossible altogether.

In sum, logistic regression provides an excellent method for effectively estimating survey-specific nest decay time with only two visits. However, it remains imperative to use a sample size that is representative of the whole nest population, and an appropriate time between visits. To best reflect the conditions to which nests are exposed, nest decay studies must start before the survey and continue throughout, with revisits taking place during or immediately after the survey [57].

Concerning sample size, Buckland et al. [36] recommended a minimum of 50 individual samples to allow reliable modelling of dung decay time using logistic regression. However, as bonobo nest decay time is not only affected by ecological parameters such as habitat, rainfall or number of storms, but also by behavioural factors such as construction type and choice of tree species, the decay time of bonobo nests shows a larger variation than that of dung. Therefore, we found that for bonobo nests, a minimum of 150 nests are needed for reliable estimates (Fig 4).

## Conclusions

The Congo region plays a key role in assessing global warming conditions. Due to the lack of real data from this region, models that prospect the impact of climate change into the future are so far contradictory [102, 103]. Here, 15 years of data collection revealed a marked decrease in yearly rainfall and differential temperature between 2003 and 2018, but a constant number of storms. As a result, most of the rain in recent years at LuiKotale has come in the form of storms. Drier conditions have resulted in longer nest decay times, suggested to be further prolonged by the building of stronger nests necessitated by rarer, but harsher and more unpredictable, precipitation. Climate change is a reality in the middle of the Congo Basin, and this trend is likely to extend across the range of great ape distribution. As climate change continues to affect both the process of nest decomposition and ape nest construction behaviour, great ape nest decomposition times are likely to increase further in future years. This will create an opportunity for the erroneous conclusion of increasing ape numbers even when populations are stable or decreasing. In conclusion, we stress the absolute necessity to obtain and apply accurate, survey-specific nest decay estimates. Failure to account for the variation of decay time both between and within sites will lead to unreliable population estimates, having serious implications for our understanding of the dynamics of great ape populations and jeopardizing the very foundations of the conservation of great apes.

## Supporting information

S1 Appendix Stan code of gamma survival model. (TXT)

S2 Appendix Results of model selection procedure. (DOCX)

S1 Table Decay time for nest constructed in tree species (categories; parameters) in this study (all), and by survey period (P1, P2). Species: Genus and species name; Vernacular name: local (Lonkundu) name of tree species; Sample: number of individual trees for each category; Parameter mean (95% CI) log-scale: posterior mean with 95% confidence Interval of each parameter *j*; Average decomposition time (95% CI) natural scale (Days) with 95% confidence interval. Category 11 (i.e. “Other species”): all other tree species, including those of integrated nests.
(DOCX)

S2 Table Studies using a logistic regression for estimating the average decay time for great apes (species) across different study areas. Sample (n): number of individual nests included in the study. Survey duration: the duration of the study in months. Revisit: time after which the nests were revisited.
(DOCX)

S3 Table Model checking and diagnostic using Pareto smoothed importance-sampling (PSIS) leave-one-out cross-validation [76]. For each model we provide 1) Number of data-points: number of individual nests; 2) Number of parameters: number of parameters estimated by the model (* = tree species’ parameters not included); 3) elpd (SE): estimated log pointwise predictive density (elpd) and relative standard error (SE), 4) p (SE): effective number of parameters (p) and relative standard error (SE); 5) looic: leave one out information criterion with relative standard error (SE); 5) Pareto k diagnostic values: reliability and approximate convergence of the PSIS-based estimates (values < 0.7 are considered acceptable [76]); 6) Monte Carlo SE of elpd: pointwise values of the Monte Carlo standard error (SE) of elpd. In S3, one data point was found having a Pareto k value > 0.7, indicating a highly influential data point, possibly biasing the estimated diagnostics. By re-running the model leaving out the influential data-point [74], we obtained reliable estimates (model “S3b”).
(DOCX)

S4 Table Decay time (mean; (95% CI) of nests assessed for different survey times of 1) dataset published by Mohneke and Fruth [73]; 2) Survey 4 (this study), using a a) Logistic regression [57] investigating different times of revisit [Proportion of nests “Alive” at revisit]); b) Gamma survival model [this study]. 1) Different survey time (n = sample size): a) Full: complete dataset, b) Half 1: first half of the survey; c) Half 2: second half of the survey. 2) Different intervals between nest marking and revisit (for logistic regression only): a) 2 weeks = 2 weeks after the last nest group was marked; b) 1 month = 1 month after the last nest group was marked; c) 3 months = 3 months after the last nest group was marked; d) 3 months after marking = 3 months after each nest group was marked, e) Random = random number of days between 7 and 360 days after each nest group was marked. Observed mean decay time for the full dataset reported by Mohneke and Fruth was 75.5 days (95% confidence interval = 68.4–82.5) [73].
(DOCX)

S5 Table Factors influencing nest decomposition time: Results of the period-specific survival models (P1 (2003–2007); P2 (2016–2018)). For each category, (parameter) included in the models we show 1) Parameter mean: posterior mean with 95% confidence interval (95% CI) (log-scale); and 2) Average decomposition time: with 95% confidence interval (95% CI) (natural scale (Days)).
(DOCX)

S1 Fig Climate in LuiKotale, Central Congo Basin, DRC (2003–2018). Left, “Average rainfall” (*W*): average rainfall per year (mm/m^2^) shows a significant decreasing trend across the years (linear regression: *R*^*2*^ = 0.363; *p* = 0.013); Top-right, “Daily number of storms” (*S*): average daily number of storms per year (*R*^*2*^ = 0.063; *p* = 0.348; NS); Bottom-right, “Differential temperature” (*D*): average differential temperature (max-min °C) per year (C°) (*R*^*2*^ = 0.248; *p* = 0.049).
(TIFF)

S2 Fig Monthly climate of a) Periods (2003–2007, P1, n = 44; 2016–2018, P2, n = 27), and b) Surveys (S1, n = 15; S2, n = 13; S3, n = 16; S4, n = 15; S5, n = 15). From top to bottom: 1) average monthly precipitation (mm); 2) Average monthly number of storms; 3) Average monthly differential temperature between night and day (C°).
(TIFF)

S3 Fig Trace-plots of the realized iterations (n = 2000) for the main model (i.e. including all nests), for all factors listed in Table 1 (main text). The chains (n = 2) always indicate good mixing and convergence. Similar results were obtained for all other models described in the study (i.e. by periods or by survey). Warmup iteration (n = 2000) are not shown.
(TIFF)

S4 Fig Nest survival curve. Survival curve including all nests (n = 1,511; blue solid line) with 95% confidence intervals (dashed blue lines). Black dots: proportion of nests still present after a certain number of days (censored nests (n = 203) included).
(TIFF)

S5 Fig Estimated mean decay time of 10 datasets randomly selected from (a) Mohneke and Fruth [73] and (b) Survey 4 “S4” [this study], using a gamma survival model. Clouds of dots represent 2,000 mean decay times extracted from the posterior distribution, with boxplots representing quartiles and variability of the samples. From left to right: 1) full datasets (green), i.e. “All”; 10 random draws of 2) 75% of nest groups (blue); 3) 25% of nest groups (yellow). The upper x axis shows the number of individual nests *n* in each draw. Green, horizontal lines show the reference decay value (mean, solid line) and 95% confidence intervals (dashed lines) for a) decay time for Mohneke and Fruth [73]; b) S4 estimated via gamma survival model [this study].
(TIFF)

S6 Fig Posterior distribution of contrasts (differences in days) between categories within the factors listed in Table 1 (main text). If the mass of the contrasts (coloured lines) overlaps 0 (dashed grey line), then the difference between mean decay times for 2 categories is not significant. 1) Blue lines (left): contrasts of binary factors. 2) Red lines (right): contrasts of three level factors (Dark red =“Low”—“High”; Red = “Medium”—“High”; Light red = “Low”- “Medium”). 3) Green lines (bottom-right): contrasts tree species (Dark green = *Scorodophloeus zenkeri*–“Other species”; Green = *Anonidium mannii*–“Other species”; Olive green = *Scorodophloeus zenkeri—Anonidium mannii)*.
(TIFF)
