# Supplementary material for: No time to rest: How the effects of climate change on nest decay threaten the conservation of apes in the wild
Source: PLoS One. 2021 Jun 30;16(6):e0252527. doi: 10.1371/journal.pone.0252527 (PMC8244864; doi:10.1371/journal.pone.0252527)
Supplement: S2 Appendix — (DOCX) [file pone.0252527.s002.docx]

Models containing differential temperature (*D*) and relative nest height (*H*) returned a better fitting than those containing minimum temperature at construction (*T*) and absolute nest height (*A*) (Table 2 – Models 1 to 4). However, although the raw average daily amount of rainfall throughout a nest’s life (*W*) and the raw average daily number of storms (*S*) were correlated (Spearman’s correlation test: *rho* = 0.74, *p* < 0.001), we did not detect signs of correlation between *W* and *S* related parameters after reclassification (S2 Appendix-Fig).

**S2 Appendix-Fig.** **Scatter plots of the relationship between the posterior distributions of the parameters for 1) Average rain (ω) and 2) Average storms (σ).**

(*S*) was the most important factor in our main model (including all nests, from both periods) (Table 2 – Models 5 to 15), with other important variables being nest exposure (*E*), nest construction type (*C*) and tree species (*SP*). All other factors added very little to the overall predictive power of the model.

| **Model** | **Model description** | **Difference in ELPD** | **Standard error** |
| --- | --- | --- | --- |
| 1 | Differential temperature *D* & absolute height *A* | -11.8 | 5.7 |
| 2 | Minimum temperature *T* & absolute height *A* | -9.4 | 5.7 |
| 3 | Minimum temperature *T* & relative height H | -6.5 | 4.4 |
| **4** | **Differential temperature *D* & relative height *H*** | **0** | **0** |
| 4.a | Model 4 – Differential temperature *D* | -0.2 | 1.7 |
| 4.b | Model 4 – Average rain *W* | -0.4 | 1.7 |
| 4.c | Model 4 – Forest type *F* | -1.1 | 1.7 |
| 4.d | Model 4 – Rain at construction *R* | -1.2 | 1.0 |
| 4.e | Model 4 – Relative height *H* | -1.9 | 2.6 |
| 4.f* | Model 4 – Nest position *P* | -2.8 | 2.5 |
| 4.g* | Model 4 – Construction type *C* | -7.2 | 3.4 |
| 4.h* | Model 4 – Species *SP* | -7.4 | 5.8 |
| 4.i* | Model 4 – Nest exposure *E* | -7.6 | 4.9 |
| 4.j* | Model 4 – Average storms *S* | -97.6 | 11.6 |
| 5 | Null model | -181.0 | 17.9 |

**S2 Appendix-Table. Differences in expected log predictive density (ELPD) between competing models and estimated standard errors of the differences.** If the difference between models is higher than the standard error, models with higher ELPD are expected to have better predictive performance. Models 1 to 4 include all variables specified in Table 1, but with different combinations of those describing nest height (*A* vs. *H*) and temperature at construction (*T* vs. *D*), with model 4, including *H* and *D*, returning the best predictive performance (in bold). ELPD differences of leaving out one variable at a time (Models 4.a to 4.j) are then compared to the full model (Model 4). If an important variable is excluded, then the ELPD difference with the full model will be higher. The average number of storms in a nest’s life is the most important factor describing nest decomposition time. Its exclusion returns the worst predictive performance, only better than the null model (5). * = variables significantly increasing the model’s predictive performance.
