# Supplementary material for: No time to rest: How the effects of climate change on nest decay threaten the conservation of apes in the wild
Source: PLoS One. 2021 Jun 30;16(6):e0252527. doi: 10.1371/journal.pone.0252527 (PMC8244864; doi:10.1371/journal.pone.0252527)
Supplement: S1 Table — Species: Genus and species name; Vernacular name: local (Lonkundu) name of tree species; Sample: number of individual trees for each category; Parameter mean (95% CI) log-scale: posterior mean with 95% confidence Interval of each parameter j; Average decomposition time (95% CI) natural scale (Days) with 95% confidence interval. Category 11 (i.e. “Other species”): all other tree species, including those of integrated nests. (DOCX) [file pone.0252527.s003.docx]

| Survey | Category | Species | Vernacular name | Sample | Parameter | Parameter mean (95% CI)  Log-scale | Average decomposition time (95% CI)  Natural scale (Days) |
| --- | --- | --- | --- | --- | --- | --- | --- |
| ALL | 1^a^ | *Dialium spp.* | Maku pembe | 383 | ψ_1_ | -0.04 (-2.87 - 2.79) | 87.39 ( 81.31 - 93.52) |
|  | 2^b^ | *Dialium spp.* | Maku rouge | 139 | ψ_2_ | -0.03 (-2.86 - 2.80) | 88.65 ( 79.25 - 99.07) |
|  | 3 | *Greenwayodendron suaveolens* | Bodzinda | 99 | ψ_3_ | 0.2 (-2.65 - 3.06) | 111.47 ( 98.43 - 125.50) |
|  | 4 | *Plagiostyles africana* | Bondenge | 48 | ψ_4_ | 0.21 (-2.62 - 3.07) | 113.1 ( 94.71 - 132.26) |
|  | 5 | *Monopetalanthus microphyllus* | Bokese | 40 | ψ_5_ | -0.05 (-2.89 - 2.79) | 86.91 ( 68.98 - 105.51) |
|  | 6 | *Scorodophloeus zenkeri* | Bopidji | 42 | ψ_6_ | -0.08 (-2.89 - 2.73) | 84.31 ( 67.60 - 102.17) |
|  | 7 | *Santiria trimera* | Botalala | 40 | ψ_7_ | 0.12 (-2.74 - 2.96) | 103.37 ( 84.55 - 124.06) |
|  | 8 | *Anonidium mannii* | Bodzingo | 34 | ψ_8_ | 0.34 (-2.51 - 3.17) | 128.93 (105.77 - 153.04) |
|  | 9 | *Cynometra sessiliflora* | Eaka | 27 | ψ_9_ | 0.23 (-2.57 - 3.04) | 114.9 ( 87.89 - 145.59) |
|  | 10 | *Gilbertiodendron dewevrei* | Bolapa | 25 | ψ_10_ | -0.02 (-2.89 - 2.78) | 89.57 ( 66.22 - 113.90) |
|  | 11 | *Other species* | | 634 | ψ_11_ | 0.05 (-2.80 - 2.89) | 96.05 ( 90.74 - 101.64) |
| P1 | 1^a^ | *Dialium spp.* | Maku pembe | 203 | ψ_1_ | -0.08 (-2.77 - 2.84) | 79.11 ( 72.49 - 86.54) |
|  | 2^b^ | *Dialium spp.* | Maku rouge | 74 | ψ_2_ | -0.08 (-2.76 - 2.85) | 79.30 ( 67.61 - 91.56) |
|  | 3 | *Greenwayodendron suaveolens* | Bodzinda | 59 | ψ_3_ | 0.19 (-2.42 - 3.16) | 104.47 ( 89.03 - 120.45) |
|  | 4 | *Monopetalanthus microphyllus* | Bokese | 27 | ψ_4_ | -0.18 (-2.86 - 2.71) | 72.64 ( 55.44 - 92.45) |
|  | 5 | *Cynometra sessiliflora* | Eaka | 24 | ψ_5_ | 0.15 (-2.52 - 3.10) | 100.77 ( 77.67 - 128.09) |
|  | 6 | *Gilbertiodendron dewevrei* | Bolapa | 21 | ψ_6_ | -0.05 (-2.67 - 2.93) | 82.80 ( 59.71 - 105.65) |
|  | 7 | *Scorodophloeus zenkeri* | Bopidji | 18 | ψ_7_ | -0.14 (-2.80 - 2.76) | 75.70 ( 54.57 - 100.22) |
|  | 8 | *Santiria trimera* | Botalala | 16 | ψ_8_ | 0.19 (-2.48 - 3.16) | 104.97 ( 79.65 - 133.60) |
|  | 9 | *Maesobotrya bertramiana* | Kalanga | 16 | ψ_9_ | -0.09 (-2.86 - 2.85) | 79.78 ( 57.20 - 105.06) |
|  | 10^c^ | *Diospyros spp.* | Mandza | 14 | ψ_10_ | -0.12 (-2.78 - 2.75) | 77.18 ( 51.44 - 105.91) |
|  | 11 | *Other species* | | 359 | ψ_11_ | 0.07 (-2.63 - 3.01) | 92.18 ( 85.59 - 98.69) |
| P2 | 1^a^ | *Dialium spp.* | Maku Pembe | 180 | ψ_1_ | -0.17 (-2.69 - 2.63) | 96.27 ( 87.13 - 106.41) |
|  | 2^b^ | *Dialium spp.* | Maku Rouge | 65 | ψ_2_ | -0.13 (-2.61 - 2.64) | 99.94 ( 84.29 - 117.23) |
|  | 3 | *Greenwayodendron suaveolens* | Bodzinda | 40 | ψ_3_ | 0.07 (-2.44 - 2.83) | 121.86 ( 99.85 - 144.63) |
|  | 4 | *Plagiostyles africana* | Bondenge | 36 | ψ_4_ | 0.08 (-2.44 - 2.91) | 123.83 (100.54 - 150.77) |
|  | 5 | *Scorodophloeus zenkeri* | Bopidji | 24 | ψ_5_ | -0.18 (-2.62 - 2.72) | 95.50 ( 72.08 - 121.39) |
|  | 6 | *Anonidium mannii* | Bodzingo | 24 | ψ_6_ | 0.10 (-2.40 - 2.91) | 126.65 ( 97.49 - 158.92) |
|  | 7 | *Santiria trimera* | Botalala | 24 | ψ_7_ | -0.03 (-2.52 - 2.82) | 111.60 ( 84.60 - 143.76) |
|  | 8^d^ | *Grewia spp.* | Bopfumo | 10 | ψ_8_ | -0.32 (-2.85 - 2.65) | 84.21 ( 52.61 - 119.40) |
|  | 9 | *Trichoscypha arborescens* | Bohungwu | 12 | ψ_9_ | -0.41 (-2.89 - 2.42) | 76.94 ( 50.99 - 107.96) |
|  | 10 | *Monopetalanthus microphyllus* | Bokese | 13 | ψ_10_ | 0.00 (-2.53 - 2.85) | 115.08 ( 78.27 - 158.39) |
|  | 11 | *Other species* | | 252 | ψ_11_ | -0.01 (-2.48 - 2.81) | 112.39 (101.93 - 123.22) |

^a^ Includes five *Dialium* species identical by vernacular name: *D. angolense; D. gossweileri; D. kasaiense; D.pachyphyllum; D. tessmannii*

^b^ Includes two *Dialium* species identical by vernacular name: *D. corbisieri; D. zenkeri*

^c^ Includes five *Diospyros* species identical by vernacular name: *D. bipendensis; D. gilletii; D. iturensis; D. melocarpa; D. zenkeri*

^d^ Includes three *Grewia* species identical by vernacular name: G. coriacea; G. oligoneura; G. pinnatifida
