# Supplementary material for: No time to rest: How the effects of climate change on nest decay threaten the conservation of apes in the wild
Source: PLoS One. 2021 Jun 30;16(6):e0252527. doi: 10.1371/journal.pone.0252527 (PMC8244864; doi:10.1371/journal.pone.0252527)
Supplement: S2 Table — Sample (n): number of individual nests included in the study. Survey duration: the duration of the study in months. Revisit: time after which the nests were revisited. (DOCX) [file pone.0252527.s004.docx]

| Species | Study area | Sample (n) | Survey duration | Revisit |
| --- | --- | --- | --- | --- |
| *Pan paniscus* | Lake Tumba; Democratic Republic of the Congo [83] | 610 | 22 months | Weekly |
| *Pan troglodytes troglodytes* | Goualougo Triangle; Republic of Congo [63] | 92 | 7 months | NA |
| *Pan troglodytes troglodytes* | Mbam Ndjerem National Park; Cameroon [64] | 309 | 15 months | 3 months after nest marking |
| *Pan troglodytes verus* | Tai National Park; Côte d'Ivoire [38] | 141 | 6 months | Weekly, until 80% nests were decayed |
| *Pan troglodytes verus* | Rio Muni Landscape; Equatorial Guinea [92] | 76 | 3 months | From 14 to 202 days after nest marking |
| *Pan troglodytes verus* | Several sites; Liberia [94] | 165 | 5 months | From 6 to 310 days after nest marking |
| *Pongo abelii* | Several sites; Sumatra, Indonesia [82] | 753 | NA | NA |
| *Pongo pygmaeus* | Sabangau and Lesan; Borneo, Indonesia [81] | 423 (Sabangau)  88 (Lesan) | 118 months (Sabangau)  20 months (Lesan) | Weekly |
