# Supplementary material for: No time to rest: How the effects of climate change on nest decay threaten the conservation of apes in the wild
Source: PLoS One. 2021 Jun 30;16(6):e0252527. doi: 10.1371/journal.pone.0252527 (PMC8244864; doi:10.1371/journal.pone.0252527)
Supplement: S3 Table — For each model we provide 1) Number of data-points: number of individual nests; 2) Number of parameters: number of parameters estimated by the model (* = tree species’ parameters not included); 3) elpd (SE): estimated log pointwise predictive density (elpd) and relative standard error (SE), 4) p (SE): effective number of parameters (p) and relative standard error (SE); 5) looic: leave one out information criterion with relative standard error (SE); 5) Pareto k diagnostic values: reliability and approximate convergence of the PSIS-based estimates (values < 0.7 are considered acceptable [76]); 6) Monte Carlo SE of elpd: pointwise values of the Monte Carlo standard error (SE) of elpd. In S3, one data point was found having a Pareto k value > 0.7, indicating a highly influential data point, possibly biasing the estimated diagnostics. By re-running the model leaving out the influential data-point [74], we obtained reliable estimates (model “S3b”). (DOCX) [file pone.0252527.s005.docx]

| Model | Number of data points | Number of parameters | elpd (SE) | p (SE) | looic (SE) | Pareto k diagnostic values | Monte Carlo SE of elpd |
| --- | --- | --- | --- | --- | --- | --- | --- |
| *MAIN* | 1,511 | 35 | -7088.8 (67.9) | 22.8 (1.0) | 14177.7 (135.8) | 1,511 < 0.5 | 0.1 |
| *P1* | 832 | 35 | -3938.4 (47.0) | 21.3 (1.1) | 7876.8 (94.0) | 832 < 0.5 | 0.1 |
| *P2* | 679 | 35 | -3138.1 (49.7) | 23.3 (1.6) | 6276.0 (99.4) | 679 < 0.5 | 0.1 |
| *S1** | 278 | 23 | -1167.1 (27.8) | 13.3 (1.9) | 2334.2 (55.5) | 278 < 0.5 | 0.1 |
| *S2** | 305 | 23 | -1446.9 (26.3) | 15.1 (1.6) | 3113.9 (52.7) | 305 < 0.5 | 0.1 |
| *S3** | 249 | 23 | -1155.3 (27.4) | 12.6 (1.5) | 2310.6 (54.8) | 248 < 0.5  1 < 1.0^§^ | NA |
| *S3b** | 248 | 23 | -1149.0 (27.4) | 11.5 (1.2) | 2298.9 (54.7) | 248 < 0.5 | 0.1 |
| *S4** | 450 | 23 | -2324.9 (27.0) | 13.6 (1.2) | 4649.8 (54.1) | 450 < 0.5 | 0.1 |
| *S5** | 229 | 23 | -806.3 (35.9) | 12.8 (1.8) | 1612.7 (71.8) | 226 < 0.5  3 < 0.7 | 0.1 |
