# Supplementary material for: No time to rest: How the effects of climate change on nest decay threaten the conservation of apes in the wild
Source: PLoS One. 2021 Jun 30;16(6):e0252527. doi: 10.1371/journal.pone.0252527 (PMC8244864; doi:10.1371/journal.pone.0252527)
Supplement: S4 Table — 1) Different survey time (n = sample size): a) Full: complete dataset, b) Half 1: first half of the survey; c) Half 2: second half of the survey. 2) Different intervals between nest marking and revisit (for logistic regression only): a) 2 weeks = 2 weeks after the last nest group was marked; b) 1 month = 1 month after the last nest group was marked; c) 3 months = 3 months after the last nest group was marked; d) 3 months after marking = 3 months after each nest group was marked, e) Random = random number of days between 7 and 360 days after each nest group was marked. Observed mean decay time for the full dataset reported by Mohneke and Fruth was 75.5 days (95% confidence interval = 68.4–82.5) [73]. (DOCX) [file pone.0252527.s006.docx]

|  |  | Logistic regression | | | | | Gamma survival |
| --- | --- | --- | --- | --- | --- | --- | --- |
| Dataset | **Survey time** | *2 weeks* | *1 month* | *3 months* | *3 months after marking* | *Random* | *NA* |
| *Mohneke & Fruth [74]* | *Full*  *(n = 218)* | 85.3  (84.0 - 89.8)  [0.243] | 78.2  (77.4 - 87.5)  [0.170] | 38.9  36.0 - 64.6  [0.004] | 82.5  (79.7 - 84.2)  [0.367] | 78.2  (77.3 - 80.6)  [0.238] | 77.7  (71.0 - 84.9)  [*NA*] |
|  | *Half 1*  *(n = 125)* | 66.4  (63.5 - 67.3)  [0.536] | 70.5  (68.2 - 71.9)  [0.448] | 86.1  (83.9 - 91.9)  [0.192] | 78.7  (78.1 - 83.2)  [0.352] | 68.1  (66.6 - 70.1)  [0.248] | 77.8  (69.9 - 86.6)  [*NA*] |
|  | *Half 2*  *(n = 93)* | 90.9  (81.8 - 94.0)  [0.419] | 76.7  (74.3 - 82.4)  [0.279] | *NA*  *NA*  [0.000] | 84.7  (81.8 - 88.1)  [0.387] | 93.8  (92.9 - 97.6)  [0.236] | 78.5  (68.1 - 90.4)  [*NA*] |
| *S4 [this study]* | *Full*  *(n = 450)* | 104.8  (103.6 - 107.5)  [0.275] | 105.4  (104.6 - 108.1)  [0.238] | 102.1  (100.6 - 112.5)  [0.111] | 96.9;  (88.5 - 98.9)  [0.464] | 102.3;  (100.6 - 109.1)  [0.293] | 104.6;  (98.7 - 110.5)  [*NA*] |
|  | *Half 1*  *(n = 221)* | 96.5  (92.5 - 98.4)  [0.412] | 91.42  (87.4 - 94.1)  [0.326] | 97.4  (94.7 - 100.6)  [0.140] | 84.4;  (82.1 - 87.0)  [0.389] | 90.8;  (87.9 - 95.5)  [0.294] | 87.2;  (77.8 - 97.7)  [*NA*] |
|  | *Half 2*  *(n = 229)* | 101.5  (100.2 - 103.1)  [0.511] | 102.4  (101.4 - 104.3)  [0.441] | 101.1  (100.3 - 109.9) [0.201] | 103.7;  (93.2 - 116.4)  [0.537] | 114.2;  (111.6 - 126.4)  [0.292] | 109.1;  (98.1 - 121.9)  [*NA*] |
