# Supplementary material for: No time to rest: How the effects of climate change on nest decay threaten the conservation of apes in the wild
Source: PLoS One. 2021 Jun 30;16(6):e0252527. doi: 10.1371/journal.pone.0252527 (PMC8244864; doi:10.1371/journal.pone.0252527)
Supplement: S5 Table — For each category, (parameter) included in the models we show 1) Parameter mean: posterior mean with 95% confidence interval (95% CI) (log-scale); and 2) Average decomposition time: with 95% confidence interval (95% CI) (natural scale (Days)). (DOCX) [file pone.0252527.s007.docx]

| Period | Factor | | Category | Parameter | Parameter mean (95% CI)  Log-scale | Average decomposition time (95% CI)  Natural scale (Days) |
| --- | --- | --- | --- | --- | --- | --- |
| P1 | Forest type (*F*) | | Swamp | ϝ_1_ | 0.22 (-6.13 - 6.71) | 63.21 (50.04 - 77.66) |
|  |  |  | *Terra* *firma* | ϝ_2_ | 0.55 (-5.78 - 7.01) | 88.04 (83.71 - 92.63) |
|  | Nest exposure (*E*) | | Close | ε_1_ | 0.49 (-6.02 - 6.95) | 80.41 (74.45 - 86.63) |
|  |  |  | Open | ε_2_ | 0.61 (-5.87 - 7.09) | 91.09 (85.64 - 96.76) |
|  | Nest position (*P*) | | Side branch | π_1_ | 0.57 (-5.93 - 6.91) | 85.41 (80.91 - 90.34) |
|  |  |  | Top | π_2_ | 0.66 (-5.87 - 6.99) | 93.63 (83.17 - 104.89) |
|  | Nest construction type (*C*) | | Single tree | γ_1_ | 0.57 (-5.88 - 6.90) | 89.5 (84.80 - 94.40) |
|  |  |  | Integrated | γ_2_ | 0.42 (-6.01 - 6.71) | 77.14 (69.43 - 85.38) |
|  | Nest relative height (*H*) | | Low | η_1_ | 0.39 (-5.15 - 5.92) | 91.61 (81.96 - 102.36) |
|  |  |  | Medium | η_2_ | 0.35 (-5.20 - 5.87) | 87.82 (82.42 - 93.41) |
|  |  |  | High | η_3_ | 0.27 (-5.27 - 5.79) | 81.22 (73.20 - 89.58) |
|  | Average precipitation (*W*) | | Low | ω_1_ | 0.38 (-5.11 - 5.81) | 90.19 (74.58 - 107.36) |
|  |  |  | Medium | ω_2_ | 0.34 (-5.17 - 5.76) | 86.24 (80.85 - 91.78) |
|  |  |  | High | ω_3_ | 0.35 (-5.21 - 5.77) | 87.32 (77.55 - 97.65) |
|  | Average storms (*S*) | | Low | σ_1_ | -0.13 (-5.48 - 5.2) | 38.57 (31.96 - 45.78) |
|  |  |  | Medium | σ_2_ | 0.84 (-4.52 - 6.15) | 102.29 (96.20 - 108.8) |
|  |  |  | High | σ_3_ | 0.47 (-4.92 - 5.78) | 70.69 (61.61 - 80.55) |
|  | Differential temperature (*D*) | | Low | δ_1_ | 0.27 (-4.92 - 5.50) | 83.16 (74.97 - 91.73) |
|  |  |  | Medium | δ_2_ | 0.34 (-4.84 - 5.58) | 89.24 (83.57 - 95.14) |
|  |  |  | High | δ_3_ | 0.29 (-4.91 - 5.54) | 84.97 (77.68 - 92.83) |
|  | Rain at construction (*R*) | | No | ρ_1_ | 0.51 (-5.88 - 7.16) | 86.42 (82.04 - 91.14) |
|  |  |  | Yes | ρ_2_ | 0.54 (-5.85 - 7.19) | 88.36 (79.46 - 97.62) |
|  | Scale parameter | | | θ | θ | *NA* |
| P2 | Forest type (*F*) | Swamp | | ϝ_1_ | 0.60 (-5.95 - 7.06) | 136.86 (111.08 - 163.97) |
|  |  | *Terra* *firma* | | ϝ_2_ | 0.34 (-6.19 - 6.76) | 104.82 ( 98.73 - 111.27) |
|  | Nest exposure (*E*) | Close | | ε_1_ | 0.45 (-5.96 - 6.89) | 96.54 ( 88.03 - 105.69) |
|  |  | Open | | ε_2_ | 0.59 (-5.86 - 7.04) | 111.41 (104.15 - 119.02) |
|  | Nest position (*P*) | Side branch | | π_1_ | 0.43 (-6.17 - 7.04) | 104.55 ( 98.39 - 111.13) |
|  |  | Top | | π_2_ | 0.67 (-5.96 - 7.27) | 132.96 (107.76 - 159.99) |
|  | Nest construction type (*C*) | Single tree | | γ_1_ | 0.67 (-5.95 - 7.04) | 112.01 (105.15 - 119.04) |
|  |  | Integrated | | γ_2_ | 0.36 (-6.28 - 6.75) | 82.05 ( 69.55 - 95.72) |
|  | Nest relative height (*H*) | Low | | η_1_ | 0.37 (-5.16 - 5.60) | 121.09 (104.64 - 139.77) |
|  |  | Medium | | η_2_ | 0.25 (-5.28 - 5.51) | 107.29 ( 99.99 - 114.84) |
|  |  | High | | η_3_ | 0.14 (-5.42 - 5.45) | 96.16 ( 83.8 - 109.50) |
|  | Average precipitation (*W*) | Low | | ω_1_ | 0.46 (-4.80 - 5.67) | 86.09 ( 70.17 - 103.55) |
|  |  | Medium | | ω_2_ | 0.72 (-4.54 - 5.97) | 110.48 (104.04 - 117.37) |
|  |  | High | | ω_3_ | 0.04 (-5.19 - 5.26) | 56.74 ( 39.16 - 77.37) |
|  | Average storms (*S*) | Low | | σ_1_ | 0.12 (-5.17 - 5.19) | 63.01 ( 50.66 - 77.05) |
|  |  | Medium | | σ_2_ | 0.75 (-4.58 - 5.82) | 117.22 (110.4 - 124.57) |
|  |  | High | | σ_3_ | 0.18 (-5.15 - 5.29) | 66.24 ( 54.42 - 79.04) |
|  | Differential temperature (*D*) | Low | | δ_1_ | 0.46 (-5.21 - 5.89) | 107.29 ( 94.63 - 121.23) |
|  |  | Medium | | δ_2_ | 0.45 (-5.20 - 5.85) | 105.96 ( 99.00 - 113.27) |
|  |  | High | | δ_3_ | 0.48 (-5.17 - 5.88) | 108.48 ( 94.36 - 123.71) |
|  | Rain at construction (*R*) | No | | ρ_1_ | 0.56 (-6.11 - 7.02) | 106.58 (100.53 - 113.02) |
|  |  | Yes | | ρ_2_ | 0.55 (-6.09 - 7.00) | 106.39 ( 89.94 - 124.60) |
|  | Scale parameter | | | θ | θ | *NA* |
